# Association between raised blood pressure and elevated serum liver enzymes among active-duty Royal Thai Army personnel in Thailand

**DOI:** 10.1186/s12872-023-03181-3

**Published:** 2023-03-21

**Authors:** Boonsub Sakboonyarat, Jaturon Poovieng, Sethapong Lertsakulbunlue, Kanlaya Jongcherdchootrakul, Phutsapong Srisawat, Mathirut Mungthin, Ram Rangsin

**Affiliations:** 1 Department of Military and Community Medicine, Phramongkutklao College of Medicine, Bangkok, 10400 Thailand; 2 Department of Medicine, Phramongkutklao College of Medicine, Bangkok, 10400 Thailand; 3 Department of Pharmacology, Phramongkutklao College of Medicine, Bangkok, 10400 Thailand; 4 Department of Parasitology, Phramongkutklao College of Medicine, Bangkok, 10400 Thailand

**Keywords:** Hypertension, Blood pressure, Liver enzymes, AST, ALT, RTA, Thailand

## Abstract

**Background:**

The relationship between hypertension (HT) and serum liver enzymes was reported in a few studies, but the findings were inconsistent. Therefore, the present study aimed to identify the association between elevated serum liver enzymes and raised BP through the use of a large sample of Royal Thai Army (RTA) personnel.

**Methods:**

The dataset obtained from the annual health examination database of RTA personnel in Thailand was utilized. A total of 244,281 RTA personnel aged 35–60 were included in the current study. Elevated serum liver enzymes were defined as aspartate aminotransferase (AST) or alanine aminotransferase (ALT) ≥ 40 U/L in males and ≥ 35 U/L in females. HT was defined as systolic BP ≥ 140 or diastolic BP ≥ 90 mmHg. A multivariable linear regression model was used to estimate the coefficient and 95% confidence intervals (CI), whereas a multivariable logistic regression model was applied to estimate adjusted odds ratios (AORs) and 95% CI for the association between raised BP and serum liver enzymes.

**Results:**

Compared to individuals with SBP < 120 and DBP < 80 mmHg, the β coefficients of log-transformed AST and ALT were 0.13 (95% CI: 0.12–0.13) and 0.11 (95% CI: 0.11–0.12) in males with HT. Meanwhile, the β  coefficients of log-transformed AST and ALT were 0.03 (95% CI: 0.02–0.04) and 0.07 (95% CI: 0.05–0.08) in females with HT. In males, HT was associated with elevated AST (AOR: 1.92; 95% CI: 1.85–2.01) and elevated ALT (AOR: 1.43; 95% CI: 1.38–1.48). On the other hand, in females, HT was associated with elevated AST (AOR: 1.42; 95% CI: 1.21–1.66) and elevated ALT (AOR: 1.38; 95% CI: 1.21–1.57).

**Conclusion:**

Raised BP was positively correlated with elevated AST and ALT in active-duty RTA personnel. Moreover, HT was independently attributed to higher odds of elevated AST and ALT in comparison to optimal BP in both males and females. Furthermore, the relationship between serum liver enzymes and BP was modified by sex.

**Supplementary Information:**

The online version contains supplementary material available at 10.1186/s12872-023-03181-3.

## Background

High blood pressure (BP) is a major cause of cardiovascular diseases (CVD) affecting more than 30% of adults worldwide [1]. Similarly, in Thailand, the National Health Examination Survey VI in 2019 demonstrated that 25% of Thai adults aged $\ge$ 15 years suffer from hypertension (HT) [2]. Raised BP is a leading cause of end-organ damage, including ischemic heart disease, stroke, and chronic kidney disease [3–7]. Furthermore, a few studies reported the relationship between HT and liver dysfunction [8, 9].

The liver is a vital organ that plays essential roles, including biomolecules' synthesis, storage, degradation, and transformation [10, 11]. The liver enzymes, consisting of aspartate aminotransferase (AST) and alanine aminotransferase (ALT), were suggested to have substantial clinical and convenient surrogate markers that reflect excess fat deposition in the liver and nonalcoholic fatty liver disease (NAFLD) and other related dysfunctions [12–14]. As mentioned above, recently, a few studies reported the connection between liver enzymes and high BP, which may occur through direct partway as insulin resistance resulting in simple steatosis and nonalcoholic steatohepatitis [10].

However, the association between liver enzymes and elevated BP has been reported in limited studies with small sample sizes, in which the findings were conflicting. For instance, a previous study on Bangladeshi adults indicated that only ALT and gamma-glutamyl transferase (GGT) not AST were related to HT [13]. At the same time, a related study in Iran expressed that after adjusting for the potential confounder, AST, ALT, and GGT were not associated with HT [9]. Nevertheless, the evidence from adults in Thailand is yet to be available.

In Thailand, nearly 50,000 active-duty Royal Thai Army (RTA) personnel aged at least 35 years participate in yearly health examinations provided by the RTA Medical Department (RTAMED). Raised BP was still a crucial health issue among this population between 2017 and 2021 [15]. Therefore, we aimed to adopt an extensive database of RTA personnel's physical health examinations from 2017 to 2021 so that we can identify the association between elevated serum liver enzymes and raised BP. Furthermore, sex-specific associations between serum liver enzymes and raised BP were assessed among this study population.

## Methods

### Study design and subjects

The current study employed the dataset obtained from the annual health examination database of RTA personnel in 2017–2021 from the RTAMED in Bangkok, Thailand [15, 16]. The RTAMED provides annual health examinations for active-duty RTA personnel through 37 RTA hospitals nationwide, the Army Institute of Pathology (AIP), and the Armed Forces Research Institute of Medical Sciences (AFRIMS). We included active-duty RTA personnel aged 35–60 who participated in annual health examinations between 2017 and 2021. In the current study, we intended to evaluate the association between blood pressure (BP) and serum liver enzymes. Therefore, individuals without records of BP and serum liver enzymes, carrying AST or ALT, were excluded.

### Data collection

The RTAMED provides health examinations for RTA personnel yearly through RTA hospitals nationwide, the AIP, and AFRIMS. A self-report using a standardized case report form was conducted during the health examination session in order to obtain characteristics data and behavioral factors, such as age, sex, smoking status, alcohol use, and exercise [15, 16]. Furthermore, the annual health examination database of RTA personnel comprised body weight, height, systolic blood pressure (SBP), and diastolic blood pressure (DBP). A trained operator conducted anthropometric measurements. BP was measured through the use of an automatic blood pressure monitor in the standardized technique following the Thai guidelines on the treatment of HT [17]. Body mass index (BMI) was calculated by weight (in kg) divided by height (in meter-squared) [16]. Mean arterial pressure (MAP) was measured by the following formula: DBP + 1/3(SBP – DBP) [18]. BP was categorized into four groups regarding Thai guidelines on the treatment of HT as follows [17]: (1) SBP < 120 and DBP < 80 mmHg, (2) SBP 120–129 or DBP 80–84 mmHg, (3) SBP 130–139 or DBP 85–89 mmHg, and (4) SBP ≥ 140 or DBP ≥ 90 mmHg. Laboratory data included AST, ALT, fasting plasma glucose (FPG), triglyceride (TG), and total cholesterol (TC). Elevated serum liver enzymes were defined as AST or ALT $\ge$ 40 U/L in males and $\ge$ 35 U/L in females [9].

### Statistical analysis

All statistical analyses were carried out using StataCorp. 2021, *Stata Statistical Software: Release 17*. College Station, TX: StataCorp LLC. Descriptive statistics were exploited for calculating the distribution of participants' characteristics. Categorical variables were presented as percentages, while continuous variables were displayed as mean, standard deviation (SD), median, and interquartile range (first quartile and third quartile). In order to assess the association between serum liver enzymes and blood pressure (BP), linear regression analysis was utilized. Due to the distribution of serum liver enzymes, the normality assumption may be violated; therefore, the log transformation was performed for serum liver enzymes to improve normality (Supplementary Fig. 1). The difference in the elevated liver enzyme ($\ge$ 40 U/L in males and $\ge$ 35 U/L in females) across baseline characteristics was compared by using the *Chi*-square test or Student’s *t*-test as appropriate. Moreover, logistic regression analysis was explored for estimating the odds ratio (ORs) and 95% confidence intervals (CIs) to determine the association between elevated liver enzyme and raised BP. The interaction was also tested to explore whether sex modifies the relationship between serum liver enzyme and BP. In order to adjust the potential confounders, sex-specific multivariable analysis was performed, which was coordinated for age, regions, BMI, smoking status, alcohol use, exercise, fasting plasma glucose, total cholesterol, triglyceride, and years. A two-sided *p*-value less than 0.05 was considered statistically significant.

Although the data in the present study were collected annually and separately, some individuals may repeatedly participate in the physical health examination, which may violate the dependence observation assumption. Therefore, we also conducted a sensitivity analysis to individually assess the association between raised BP and elevated serum liver enzymes each year.

### Ethics consideration

This study was reviewed and approved by the Institutional Review Board, Royal Thai Army Medical Department, following international guidelines including the Declaration of Helsinki, the Belmont Report, CIOMS Guidelines, and the International Conference on Harmonization of Technical Requirements for Registration of Pharmaceuticals for Human Use–Good Clinical Practice (ICH–GCP) (approval number S067h/64 & S056h/65). Due to the use of secondary data, a waiver of documentation of informed consent was utilized. The Institutional Review Board, Royal Thai Army Medical Department, approved an informed consent waiver.

## Results

### Characteristics of study participants

Table 1 presents the characteristics of 244,281 active-duty RTA personnel included in the study population between 2017 and 2021. The majority (about 90%) were males. The mean age of study participants ranged from 46.7 ± 7.7 years to 48.0 ± 7.1 years. Nearly, two-thirds of study participants were current drinkers, while approximately one-fourth were current smokers. The mean SBP of study participants was 130.5 ± 16.9 mmHg in 2017 and increased continuously to 132.2 ± 17.2 mmHg in 2021. However, the mean DBP ranged from 80.8 ± 11.6 mmHg to 81.4 ± 11.6 mmHg over five years. Mean AST ranged from 29.7 ± 25.9 U/L to 30.8 ± 25.7 U/L between 2017 and 2021, while ALT ranged from 31.8 ± 26.4 U/L to 35.4 ± 27.4 U/L over five years.

**Table 1:** Characteristics of study participants

Year	2017	2018	2019	2020	2021
**Characteristics**	***n* = 42,617**	***n* = 47,868**	***n* = 54,196**	***n* = 54,133**	***n* = 45,467**
**Sex, n (%)**					
Male	38,614 (90.6)	42,630 (89.1)	48,553 (89.6)	47,682 (88.1)	41,012 (90.2)
Female	4003 (9.4)	5238 (10.9)	5643 (10.4)	6451 (11.9)	4455 (9.8)
**Age (years)**					
Mean ± SD	48.0 ± 7.1	47.5 ± 7.3	47.4 ± 7.5	47.4 ± 7.7	46.7 ± 7.7
Median (Q1-Q3)	49 (42—54)	48 (41—54)	48 (41—54)	47 (40—55)	46 (40—54)
**Regions**					
Bangkok	7315 (17.2)	9730 (20.3)	10,840 (20.0)	11,085 (20.5)	5544 (12.2)
Central	15,263 (35.8)	18,024 (37.7)	19,567 (36.1)	20,899 (38.6)	18,352 (40.4)
Northeast	8271 (19.4)	7478 (15.6)	8945 (16.5)	9907 (18.3)	7881 (17.3)
North	9953 (23.4)	7432 (15.5)	9586 (17.7)	6872 (12.7)	8650 (19.0)
South	1815 (4.3)	5204 (10.9)	5258 (9.7)	5370 (9.9)	5040 (11.1)
**Current smokers**, n (%)	10,132 (24.1)	12,618 (26.7)	14,155 (26.8)	14,851 (28.8)	12,838 (28.3)
**Current alcohol use**, n (%)	27,318 (64.8)	30,063 (63.5)	34,861 (64.6)	34,787 (67.4)	28,733 (63.3)
**Exercise**, n (%)	39,168 (91.9)	44,675 (93.3)	51,086 (94.3)	50,834 (93.9)	41,482 (91.2)
**Systolic BP (mmHg)**					
Mean ± SD	130.5 ± 16.9	130.7 ± 17.0	131.0 ± 16.8	131.3 ± 16.6	132.2 ± 17.2
Median (Q_1_-Q_3_)	130 (120—140)	130 (120—140)	130 (120—140)	130 (120—140)	131 (121—140)
**Diastolic BP (mmHg)**					
Mean ± SD	81.4 ± 11.6	81.3 ± 11.7	81.0 ± 11.6	80.8 ± 11.6	81.3 ± 11.9
Median (Q_1_-Q_3_)	80 (73—89)	81 (73—89)	80 (73—88)	80 (73—88)	81 (73—89)
**AST (U/L)**					
Mean ± SD	30.8 ± 25.7	30.0 ± 24.9	29.7 ± 25.9	29.9 ± 25.5	30.0 ± 27.5
Median (Q_1_-Q_3_)	25 (21—32)	25 (20—32)	24 (20—31)	24 (20—31)	25 (20—31)
**ALT (U/L)**					
Mean ± SD	35.4 ± 27.4	34.7 ± 26.6	33.9 ± 26.2	31.8 ± 26.4	33.3 ± 27.8
Median (Q_1_-Q_3_)	29 (19—44)	28 (19—42)	27 (18—41)	25 (18—37)	26 (18—39)

*BP* Blood pressure, *SD* Standard deviation,* Q*_*1*_ Quartile 1, and *Q*_*3*_ Quartile 3

### Association between raised blood pressure and elevated serum liver enzymes

Effect modification by sex on the association between serum liver enzymes and BP was observed. Table 2 illustrates a sex-specific multivariable linear regression analysis of aminotransferase and blood pressure. A positive relationship was observed between log-transformed AST and SBP, DBP, and MAP in both males and females, with a *p*-value < 0.001. In addition, the association between log-transformed ALT and SBP, DBP, and MAP among males and females was also marked, with a *p*-value < 0.001. Consistently, when BP was further assessed as categories, in comparison with the reference group (SBP < 120 and DBP < 80 mmHg), the coefficients of log-transformed AST and ALT were 0.13 (95% CI: 0.12–0.13) and 0.11 (95% CI: 0.11–0.12) in males with SBP ≥ 140 or DBP ≥ 90. Meanwhile, the coefficients of log-transformed AST and ALT were 0.03 (95% CI: 0.02–0.04) and 0.07 (95% CI: 0.05–0.08) in females with SBP ≥ 140 or DBP ≥ 90 mmHg.

**Table 2:** Univariable and multivariable linear regression analysis for the association between raised blood pressure and serum liver enzymes

Blood pressure	Log-transformed AST			Log-transformed ALT		
	${\varvec{\beta}}$ **coefficient**	**95% CI**	***p*-value**	${\varvec{\beta}}$ **coefficient**	**95% CI**	***p*-value**
**Male**						
**SBP (mmHg)** ^**¥**§^						
Crude	0.003	0.003–0.003	< 0.001	0.004	0.003–0.004	< 0.001
Adjusted^a^	0.003	0.003–0.003	< 0.001	0.002	0.002–0.002	< 0.001
**DBP (mmHg)** ^§^						
Crude	0.005	0.005–0.005	< 0.001	0.007	0.007–0.007	< 0.001
Adjusted^a^	0.004	0.004–0.005	< 0.001	0.004	0.004–0.004	< 0.001
**MAP (mmHg)** ^**¥**§^						
Crude	0.005	0.005–0.005	< 0.001	0.006	0.006–0.006	< 0.001
Adjusted^a^	0.004	0.004–0.005	< 0.001	0.004	0.003–0.004	< 0.001
**Blood pressure (mmHg)** ^**¥**§^						
Crude						
SBP < 120 and DBP < 80	Ref			Ref		
SBP 120–129 or DBP 80–84	0.03	0.02–0.03	< 0.001	0.06	0.06–0.07	< 0.001
SBP 130–139 or DBP 85–89	0.06	0.05–0.06	< 0.001	0.11	0.10–0.11	< 0.001
SBP ≥ 140 or DBP ≥ 90	0.14	0.14–0.14	< 0.001	0.19	0.19–0.19	< 0.001
Adjusted^a^						
SBP < 120 and DBP < 80						
SBP 120–129 or DBP 80–84	0.03	0.02–0.03	< 0.001	0.04	0.03–0.04	< 0.001
SBP 130–139 or DBP 85–89	0.06	0.05–0.06	< 0.001	0.07	0.06–0.07	< 0.001
SBP ≥ 140 or DBP ≥ 90	0.13	0.12–0.13	< 0.001	0.11	0.11–0.12	< 0.001
**Female**						
**SBP (mmHg)** ^**¥**§^						
Crude	0.002	0.002–0.003	< 0.001	0.005	0.005–0.006	< 0.001
Adjusted^a^	0.001	0.001–0.001	0.001	0.001	0.001–0.002	< 0.001
**DBP (mmHg)**^§^						
Crude	0.003	0.002–0.003	< 0.001	0.008	0.007–0.009	< 0.001
Adjusted^a^	0.001	0.001–0.001	0.003	0.002	0.001–0.003	< 0.001
**MAP (mmHg)** ^**¥**§^						
Crude	0.003	0.003–0.003	< 0.001	0.008	0.008–0.009	< 0.001
Adjusted^a^	0.001	0.001–0.001	0.001	0.002	0.001–0.003	< 0.001
**Blood pressure (mmHg)** ^**¥**§^						
Crude						
SBP < 120 and DBP < 80	Ref			Ref		
SBP 120–129 or DBP 80–84	0.05	0.03–0.05	< 0.001	0.12	0.10–0.13	< 0.001
SBP 130–139 or DBP 85–89	0.07	0.06–0.08	< 0.001	0.19	0.17–0.20	< 0.001
SBP ≥ 140 or DBP ≥ 90	0.12	0.10–0.12	< 0.001	0.26	0.24–0.28	< 0.001
Adjusted^a^						
SBP < 120 and DBP < 80	Ref			Ref		
SBP 120–129 or DBP 80–84	0.01	0.001–0.020	0.013	0.04	0.02–0.05	< 0.001
SBP 130–139 or DBP 85–89	0.02	0.01–0.03	0.001	0.06	0.04–0.07	< 0.001
SBP ≥ 140 or DBP ≥ 90	0.03	0.02–0.04	< 0.001	0.07	0.05–0.08	< 0.001

*SBP* Systolic blood pressure, *DBP* Diastolic blood pressure, *MAP* Mean arterial pressure, and *CI* Confidence interval

^a^Adjusting for age, regions, body mass index, smoking status, alcohol use, exercise, fasting plasma glucose, total cholesterol, triglyceride, and years

^§^P for interaction < 0.05 (sex as an effect modifier on the association between BP and log-transformed AST)

^¥^P for interaction < 0.05 (sex as an effect modifier on the association between BP and log-transformed ALT)

**Fig. 1:**
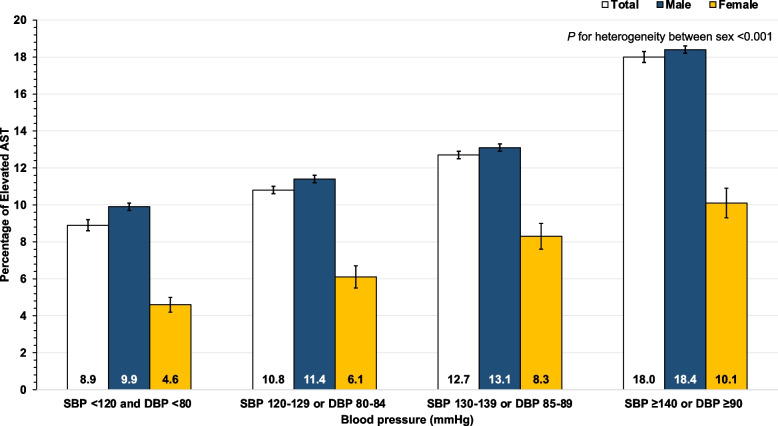
Sex-specific percentage and 95% confidence interval of elevated aspartate aminotransferase (AST) stratified by blood pressure category

**Fig. 2:**
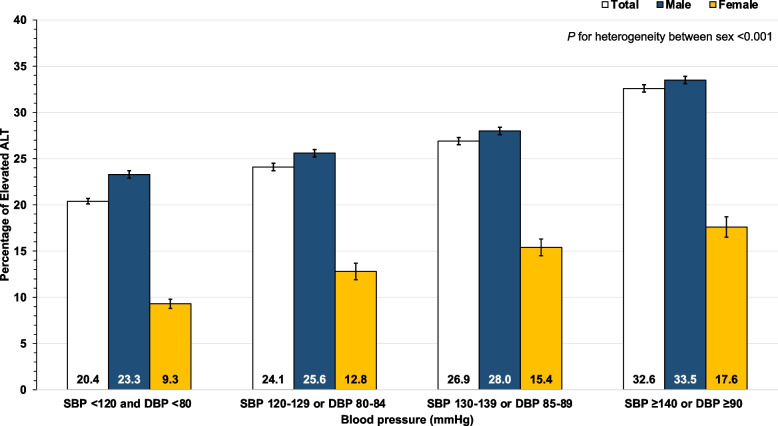
Sex-specific percentage and 95% confidence interval of elevated alanine aminotransferase (ALT) stratified by blood pressure category

Table 3 presents the association between elevated serum liver enzymes (AST or ALT ≥ 40 U/L in males and ≥ 35 U/L in females) and covariates. A higher percentage of elevated AST and ALT with higher BP was observed in both males and females (Figs. 1 and 2); furthermore, sex is the modifier of the association between raised BP and elevated serum liver enzymes (*p *for heterogeneity < 0.001). The relationship between raised BP and elevated liver enzymes was analyzed through the use of multivariable logistic regression (Table 4). After adjusting for the potential confounders, the association between BP (SBP, DBP, and MAP) and elevated aminotransferase was noticed. In males, HT (SBP ≥ 140 or DBP ≥ 90 mmHg) was associated with elevated AST (adjusted OR: 1.92; 95% CI: 1.85–2.01) and elevated ALT (adjusted OR: 1.43; 95% CI: 1.38–1.48). In females, HT (SBP ≥ 140 or DBP ≥ 90 mmHg) was associated with elevated AST (adjusted OR: 1.42; 95% CI: 1.21–1.66) and elevated ALT (adjusted OR: 1.38; 95% CI: 1.21–1.57). The results of the sensitivity analysis were presented in Supplementary Tables 1 and 2. The annual sensitivity analysis revealed that the association between raised BP and elevated serum liver enzymes followed the same pattern as the primary analysis.

**Table 3:** Relationship between baseline characteristics and elevated aminotransferase

Blood pressure	Male						Female					
	**AST < 40 U/L**	**AST ≥ 40 U/L**	***p*-value**	**ALT < 40 U/L**	**ALT ≥ 40 U/L**	***p*-value**	**AST < 35 U/L**	**AST ≥ 35 U/L**	***p*-value**	**ALT < 35 U/L**	**ALT ≥ 35 U/L**	***p*-value**
**Age (years)**												
Mean ± SD	47.4 ± 7.5	47 ± 7.4	< 0.001	47.8 ± 7.5	46.2 ± 7.3	< 0.001	47.3 ± 7.6	49.4 ± 7.3	< 0.001	47.3 ± 7.6	48.6 ± 7.5	< 0.001
**Regions**			< 0.001			< 0.001			< 0.001			< 0.001
Bangkok	31,607 (90.6)	3269 (9.4)		27,810 (79.7)	7066 (20.3)		9139 (94.8)	499 (5.2)		8822 (91.5)	816 (8.5)	
Central	71,642 (85.1)	12,519 (14.9)		62,865 (74.7)	21,296 (25.3)		7300 (91.9)	644 (8.1)		7105 (89.4)	839 (10.6)	
Northeast	32,454 (84.0)	6162 (16.0)		28,560 (74.0)	10,056 (26.0)		3609 (93.4)	257 (6.6)		3457 (89.4)	409 (10.6)	
North	33,456 (85.3)	5785 (14.7)		21,431 (54.6)	17,810 (45.4)		3037 (93.4)	215 (6.6)		2162 (66.5)	1090 (33.5)	
South	18,736 (86.8)	2861 (13.2)		15,609 (72.3)	5988 (27.7)		997 (91.5)	93 (8.5)		953 (87.4)	137 (12.6)	
**Current smokers**, n (%)			< 0.001			< 0.001			0.050			0.717
No	130,770 (87.4)	18,840 (12.6)		108,267 (72.4)	41,343 (27.6)		23,428 (93.4)	1652 (6.6)		21,878 (87.2)	3202 (12.8)	
Yes	53,037 (82.7)	11,069 (17.3)		44,402 (69.3)	19,704 (30.7)		445 (91.2)	43 (8.8)		423 (86.7)	65 (13.3)	
**Current alcohol use**, n (%)			< 0.001			< 0.001			0.673			0.016
No	58,948 (88.7)	7481 (11.3)		48,337 (72.8)	18,092 (27.2)		17,122 (93.4)	1206 (6.6)		15,931 (86.9)	2397 (13.1)	
Yes	125,957 (84.8)	22,564 (15.2)		105,270 (70.9)	43,251 (29.1)		6754 (93.3)	487 (6.7)		6375 (88.0)	866 (12.0)	
**Exercise**, n (%)			0.206			0.024			0.630			0.327
No	12,797 (85.7)	2144 (14.3)		10,807 (72.3)	4134 (27.7)		1951 (93.1)	144 (6.9)		1842 (87.9)	253 (12.1)	
Yes	175,098 (86.0)	28,452 (14.0)		145,468 (71.5)	58,082 (28.5)		22,131 (93.4)	1564 (6.6)		20,657 (87.2)	3038 (12.8)	
**Body mass index (kg/m2)**												
Mean ± SD	25.3 ± 3.6	25.5 ± 4.1	< 0.001	25.0 ± 3.5	26.4 ± 3.9	< 0.001	24.3 ± 4.1	25.8 ± 4.7	< 0.001	24.2 ± 4.1	25.9 ± 4.4	< 0.001
**Fasting plasma glucose (mg/dL)**												
Mean ± SD	103.6 ± 36.0	109.7 ± 40.9	< 0.001	102.8 ± 35.8	108.9 ± 39	< 0.001	95.7 ± 26.1	106.5 ± 37.9	< 0.001	95.1 ± 25.4	107.3 ± 37.4	< 0.001
**Total cholesterol (mg/dL)**												
Mean ± SD	211.0 ± 48.7	211.3 ± 55.5	0.263	209.1 ± 48.2	215.9 ± 52.8	< 0.001	208.2 ± 43.9	209.6 ± 46.5	0.209	207.6 ± 43.7	212.9 ± 46.9	< 0.001
**Triglyceride (mg/dL)**												
Mean ± SD	165.7 ± 121.2	223.5 ± 198.4	< 0.001	158.7 ± 117.6	211.7 ± 168.5	< 0.001	110.9 ± 65.8	144.1 ± 93.5	< 0.001	109.5 ± 65.9	137.7 ± 79.8	< 0.001
**Systolic BP (mmHg)**												
Mean ± SD	131.4 ± 16.4	136.1 ± 18.0	< 0.001	131.4 ± 16.5	133.9 ± 17.0	< 0.001	123.0 ± 16.5	127.9 ± 17.1	< 0.001	122.9 ± 16.5	126.4 ± 16.8	< 0.001
**Diastolic BP (mmHg)**												
Mean ± SD	81.4 ± 11.4	84.9 ± 12.3	< 0.001	81.1 ± 11.4	83.7 ± 11.9	< 0.001	74.8 ± 10.6	77.5 ± 11.4	< 0.001	74.6 ± 10.6	77.7 ± 11.1	< 0.001
**MAP (mmHg)**												
Mean ± SD	98.1 ± 12.1	102 ± 13.3	< 0.001	97.9 ± 12.2	100.5 ± 12.7	< 0.001	90.9 ± 11.6	94.3 ± 12.2	< 0.001	90.7 ± 11.5	93.9 ± 11.9	< 0.001
**Blood pressure (mmHg)**			< 0.001			< 0.001			< 0.001			< 0.001
SBP < 120 and DBP < 80	36,063 (90.1)	3980 (9.9)		30,721 (76.7)	9322 (23.3)		9682 (95.4)	467 (4.6)		9201 (90.7)	948 (9.3)	
SBP 120–129 or DBP 80–84	41,552 (88.6)	5369 (11.4)		34,917 (74.4)	12,004 (25.6)		5672 (93.9)	367 (6.1)		5269 (87.2)	770 (12.8)	
SBP 130–139 or DBP 85–89	49,347 (86.9)	7465 (13.1)		40,931 (72.0)	15,881 (28.0)		4852 (91.7)	437 (8.3)		4474 (84.6)	815 (15.4)	
SBP ≥ 140 or DBP ≥ 90	60,933 (81.6)	13,782 (18.4)		49,706 (66.5)	25,009 (33.5)		3876 (89.9)	437 (10.1)		3555 (82.4)	758 (17.6)	

*SBP* Systolic blood pressure, *DBP* Diastolic blood pressure, *MAP* Mean arterial pressure, and *SD* Standard deviation

**Table 4:** Univariable and multivariable logistic regression analysis for the association between raised blood pressure and elevated aminotransferase

Blood pressure	Elevated AST			Elevated ALT		
	**Odds ratio**	**95% CI**	***p*-value**	**Odds ratio**	**95% CI**	***p*-value**
**Male**						
**SBP (mmHg)** ^**¥**^						
Unadjusted model	1.02	1.02–1.02	< 0.001	1.01	1.01–1.01	< 0.001
Adjusted model^a^	1.02	1.01–1.02	< 0.001	1.01	1.01–1.01	< 0.001
**DBP (mmHg)** ^**¥**^						
Unadjusted model	1.03	1.02–1.03	< 0.001	1.02	1.02–1.02	< 0.001
Adjusted model^a^	1.02	1.02–1.02	< 0.001	1.01	1.01–1.01	< 0.001
**MAP (mmHg)** ^**¥**^						
Unadjusted model	1.02	1.02–1.03	< 0.001	1.02	1.02–1.02	< 0.001
Adjusted model^a^	1.02	1.02–1.02	< 0.001	1.01	1.01–1.01	< 0.001
**Blood pressure (mmHg)** ^**¥**§^						
Unadjusted model						
SBP < 120 and DBP < 80	Ref			Ref		
SBP 120–129 or DBP 80–84	1.17	1.12–1.22	< 0.001	1.13	1.10–1.17	< 0.001
SBP 130–139 or DBP 85–89	1.37	1.32–1.43	< 0.001	1.28	1.24–1.32	< 0.001
SBP ≥ 140 or DBP ≥ 90	2.05	1.97–2.13	< 0.001	1.66	1.61–1.70	< 0.001
Adjusted model^a^§						
SBP < 120 and DBP < 80	Ref			Ref		
SBP 120–129 or DBP 80–84	1.17	1.11–1.22	< 0.001	1.09	1.05–1.12	< 0.001
SBP 130–139 or DBP 85–89	1.35	1.30–1.42	< 0.001	1.22	1.18–1.26	< 0.001
SBP ≥ 140 or DBP ≥ 90	1.92	1.85–2.01	< 0.001	1.43	1.38–1.48	< 0.001
**Female**						
**SBP (mmHg)** ^**¥**^						
Unadjusted model	1.02	1.01–1.02	< 0.001	1.01	1.01–1.01	< 0.001
Adjusted model^a^	1.01	1.01–1.01	0.006	1.01	1.00–1.01	< 0.001
**DBP (mmHg)** ^**¥**^						
Unadjusted model	1.02	1.02–1.03	< 0.001	1.03	1.02–1.03	< 0.001
Adjusted model^a^	1.01	1.01–1.01	0.001	1.01	1.01–1.02	< 0.001
**MAP (mmHg)** ^**¥**^						
Unadjusted model	1.02	1.02–1.03	< 0.001	1.02	1.02–1.03	< 0.001
Adjusted model^a^	1.01	1.01–1.01	0.001	1.01	1.01–1.01	< 0.001
**Blood pressure (mmHg)** ^**¥**§^						
Crude model						
SBP < 120 and DBP < 80	Ref			Ref		
SBP 120–129 or DBP 80–84	1.34	1.17–1.54	< 0.001	1.42	1.28–1.57	< 0.001
SBP 130–139 or DBP 85–89	1.87	1.63–2.14	< 0.001	1.77	1.60–1.95	< 0.001
SBP ≥ 140 or DBP ≥ 90	2.34	2.04–2.68	< 0.001	2.07	1.87–2.29	< 0.001
Adjusted model^a^						
SBP < 120 and DBP < 80	Ref			Ref		
SBP 120–129 or DBP 80–84	1.12	0.97–1.30	0.135	1.22	1.09–1.38	0.001
SBP 130–139 or DBP 85–89	1.34	1.16–1.55	< 0.001	1.36	1.20–1.54	< 0.001
SBP ≥ 140 or DBP ≥ 90	1.42	1.21–1.66	< 0.001	1.38	1.21–1.57	< 0.001

*SBP* Systolic blood pressure, *DBP* Diastolic blood pressure, *MAP* Mean arterial pressure, and *CI* Confidence interval

^a^Adjusting for age, regions, body mass index, smoking status, alcohol use, exercise, fasting plasma glucose, total cholesterol, triglyceride, and years

^§^P for interaction < 0.05 (sex as an effect modifier on the assocation between BP and elevated AST)

^¥^P for interaction < 0.05 (sex as an effect modifier on the association between BP and elevated ALT)

## Discussion

The associations between raised blood pressure and elevated serum liver enzymes in active-duty RTA personnel in Thailand were identified using a large database of RTA personnel's physical health examinations. After adjusting for baseline covariates, the associations between raised BP, encompassing SBP, DBP, and MAP, and elevated serum liver enzymes, both AST and ALT, were detected. Moreover, it was also found that the odds of elevated AST and ALT were higher in RTA personnel with HT (SBP ≥ 140 or DBP ≥ 90 mmHg) than those with optimal BP (SBP < 120 and DBP < 80 mmHg) in both males and females. To the best of our knowledge, this is the first and largest study examining the relationship between high BP and elevated serum liver enzymes in the Thai population.

In line with the existing literature, the evidence of the associations between raised BP and elevated serum liver enzyme was incompatible. For instance, a small sample size study on Bangladeshi adults exposed the associations between HT and serum liver enzymes, incorporating ALT and GGT, but not AST and alkaline phosphatase (ALP). Conversely, alcohol use behavior was not included in the final model of the Bangladesh study [13]. At the same time, a related study on mild dyslipidemia participants from Korea illustrated that only GGT was associated with higher SBP and DBP [8]. On the contrary, a recent relatively large study in Iran reported that after adjusting for potential confounders, ALP was interrelated with HT in both males and females, while there were no significant associations of AST, ALT, and GGT with HT [9]. Nevertheless, in the current study, considering the secondary database, there was no chance to evaluate the linkage of ALP and GGT with BP. We reported the independent association of log-transformed AST and ALT with increased BP, comprising SBP, DBP, and MAP, among males and females. Likewise, a recent study in Iran consistently exhibited the positive association of log-transformed AST, ALT, and ALP with increasing BP in both sexes [9].

Moreover, we noticed a dose–response relationship with a relatively precise association between raised BP and elevated AST and ALT. After adjusting for baseline variables, we found that the odds for elevated AST and ALT among male RTA personnel with HT were estimated to be 92% and 43% higher than those with optimal BP. Similarly, among female participants, the odds for elevated AST and ALT in individuals with HT were estimated to be 1.42 and 1.38 times higher than those with optimal BP. In the present study, the existing potential confounders were included in the final model. Nonetheless, there is a possibility that unmeasured confounders, involving the information on antihypertensive drug use, the number of drugs used and their types, and other medications for treating their comorbidities, such as dyslipidemia and diabetes, may have an impact on the results of the study.

HT is well-documented to be associated with metabolic syndrome and hyperinsulinemia, which are the key pathways for developing simple steatosis and fatty liver [10, 19]. The most common laboratory-based test reflecting these abnormalities was the elevations in AST and ALT [10]. Our study revealed that HT was independently connected with elevated AST and ALT, which the related evidence in the animal model [20] and clinical study [21] can explain. The animal model suggested that the renin-angiotensin system (RAS), especially angiotensin II (Ang II), played a vital role in activating hepatic stellate cells for liver fibrosis [20, 22]. Moreover, the related clinical study in China manifested that Ang II level was an independent risk factor for patients with NAFLD [21]. Furthermore, Ang II type 1 receptor antagonists can alleviate this progression [23]. On the other hand, HT and elevated serum liver enzymes may be linked by oxidative stress and reactive oxygen species, which play a crucial role in the pathogenesis of HT and also affect the hepatocyte resulting in hepatocellular injuries [24, 25].

A few studies reported the sex-specific association between blood pressure and serum liver enzymes [9, 13, 26]. However, the formal test for an existing interaction in those studies was limited. The present study also found a significant effect modification between sex and BP on elevated liver enzymes. In comparison with females, males showed a stronger association between raised BP and elevated AST and ALT levels. At the same time, contradictory findings from a different study reported that the association between raised DBP and elevated AST was stronger in females, though the association between raised DBP and elevated ALT was stronger in males [9]. Estradiol has an antioxidant effect in females, which may impact serum liver enzyme levels [27, 28]. Thus, one possible mechanism for sex-specific differences in the junction between raised BP and serum liver enzymes could be the effect of sex hormones [26, 27]. However, this concept requires further investigation.

The current study encountered several limitations. Firstly, this was a cross-sectional study; the causal relationship between exposure and outcome could not be presented. Secondly, concerning the secondary database used, we did not have an opportunity to investigate the relationship between raised BP and other serum liver enzymes, containing GGT and ALP. Thirdly, the information on hepatotoxic drug uses and viral hepatitis infection, possibly affecting serum liver enzymes, was not collected; hence, some unmeasured confounders were not included in the adjusted model. Fourthly, although alcohol use was adjusted in the final model, the information on the amount and frequency of alcohol consumption was limited. Thus, residual confounding on the association between BP and elevated liver enzymes may exist. In addition, the information on waist circumference is limited; thus, central obesity, a feature of metabolic syndrome related to high BP and fatty liver, was not included in the final model. Yet, the present study regulated BMI in the multivariable analysis. Next, because the data in the present study were collected each year separately, some individuals may participate in the physical health examination more than once, which may go against the dependent observation assumption. Notwithstanding, the primary analysis results were not altered by the association between raised BP and elevated serum liver enzymes annually obtained from sensitivity analysis. Finally, the current study was carried out among active-duty RTA personnel and comprised a greater proportion of male participants; however, the results reflected an actual situation in this study population.

In addition, the present study encompassed remarkable strengths, combining a large sample size with an adjustment for potential confounders, so that the independent relationship could be assessed. Therefore, our data provided robust evidence supporting the independent association between raised BP and elevated serum liver enzymes, especially AST and ALT. Our results suggest that monitoring serum liver enzyme, a convenient surrogate marker that reflects excess fat deposition in the liver and other related dysfunctions, should be performed, particularly in individuals with raised BP.

## Conclusion

Raised BP was positively associated with elevated AST and ALT in active-duty RTA personnel. In addition, HT was independently associated with higher odds of elevated AST and ALT in comparison with optimal BP in both males and females. It was found that the relationship between serum liver enzymes and BP was modified by sex. These findings supported the evidence of the relationship between BP and serum liver enzymes.

## Supplementary Information

**Additional file 1: Figure S1.** The log transformation was performed for serum liver enzymes to improve normality. **Table S1.** Multivariable logistic regression analysis for association between raised blood pressure and elevated aminotransferase in male participant, by year. **Table S2.** Multivariable logistic regression analysis for association between raised blood pressure and elevated aminotransferase in female participant, by year.

## Data Availability

The data that support the findings of this study are available from the Royal Thai Army Medical Department, but restrictions apply to the availability of these data, which were used under license for the current study, and so are not publicly available. Data are however available from the authors upon reasonable request and with permission of the Royal Thai Army Medical Department (contact Boonsub Sakboonyarat via boonsub1991@pcm.ac.th).

## Abbreviations

BP: Blood pressure
SBP: Systolic blood pressure
DBP: Diastolic blood pressure
MAP: Mean arterial pressure
AST: Aspartate aminotransferase
ALT: Alanine aminotransferase
GGT: Gamma-glutamyl transferase
ALP: Alkaline phosphatase
NAFLD: Nonalcoholic fatty liver disease
RTA: The Royal Thai Army
RTAMED: The Royal Thai Army Medical Department
AOR: Adjusted odds ratio
CI: Confidence interval
SD: Standard deviation
