# Supplementary material for: Association between raised blood pressure and elevated serum liver enzymes among active-duty Royal Thai Army personnel in Thailand
Source: BMC Cardiovasc Disord. 2023 Mar 21;23:143. doi: 10.1186/s12872-023-03181-3 (PMC10029162; doi:10.1186/s12872-023-03181-3)
Supplement: Supplementary file 1 — Additional file 1: Figure S1. The log transformation was performed for serum liver enzymes to improve normality. Table S1. Multivariable logistic regression analysis for association between raised blood pressure and elevated aminotransferase in male participant, by year. Table S2. Multivariable logistic regression analysis for association between raised blood pressure and elevated aminotransferase in female participant, by year. [file 12872_2023_3181_MOESM1_ESM.docx]

**Appendix**

**Association between Raised Blood Pressure and Elevated Serum Liver Enzymes among Active-Duty Royal Thai Army Personnel in Thailand**

Boonsub Sakboonyarat^1^, Jaturon Poovieng^2^, Sethapong Lertsakulbunlue^3^, Kanlaya Jongcherdchootrakul^1^, Phutsapong Srisawat^1^, Mathirut Mungthin^4^, *Ram Rangsin^1^

^1^Department of Military and Community Medicine, Phramongkutklao College of Medicine, Bangkok 10400, Thailand

^2^Department of Medicine, Phramongkutklao College of Medicine, Bangkok 10400, Thailand

^3^Department of Pharmacology, Phramongkutklao College of Medicine, Bangkok 10400, Thailand

^4^Department of Parasitology, Phramongkutklao College of Medicine, Bangkok 10400, Thailand

**Table of contents**

| Figure S1. The log transformation was performed for serum liver enzymes to improve normality. | Page 3 |
| --- | --- |
| Table S1. Multivariable logistic regression analysis for association between raised blood pressure and elevated aminotransferase in male participant, by year | Page 4 |
| Table S2. Multivariable logistic regression analysis for association between raised blood pressure and elevated aminotransferase in female participant, by year | Page 5 |

**Figure S1.** The log transformation was performed for serum liver enzymes to improve normality.

| **Histogram of serum liver enzymes** | **Histogram of log-transformed serum liver enzymes** |
| --- | --- |
| **AST**  **** | **Log-transformed AST**  **** |
| **ALT**  **** | **Log-transformed ALT**  **** |

**Table S1.** Multivariable logistic regression analysis for association between raised blood pressure and elevated aminotransferase in male participants, by year

| **Blood pressure** | **Elevated AST** | | | **Elevated ALT** | | |
| --- | --- | --- | --- | --- | --- | --- |
|  | **AOR** | **95% CI** | ***p*-value** | **AOR** | **95% CI** | ***p*-value** |
| **Male** |  |  |  |  |  |  |
| **SBP (mmHg)** |  |  |  |  |  |  |
| Overall (2017-2021)* | 1.02 | 1.01-1.02 | <0.001 | 1.01 | 1.01-1.01 | <0.001 |
| 2017** | 1.01 | 1.01-1.02 | <0.001 | 1.01 | 1.01-1.01 | <0.001 |
| 2018** | 1.01 | 1.01-1.02 | <0.001 | 1.01 | 1.01-1.01 | <0.001 |
| 2019** | 1.02 | 1.02-1.02 | <0.001 | 1.01 | 1.01-1.01 | <0.001 |
| 2020** | 1.02 | 1.01-1.02 | <0.001 | 1.01 | 1.01-1.01 | <0.001 |
| 2021** | 1.02 | 1.01-1.02 | <0.001 | 1.01 | 1.01-1.01 | <0.001 |
| **DBP (mmHg)** |  |  |  |  |  |  |
| Overall (2017-2021)* | 1.02 | 1.02-1.02 | <0.001 | 1.01 | 1.01-1.01 | <0.001 |
| 2017** | 1.02 | 1.02-1.03 | <0.001 | 1.01 | 1.01-1.01 | <0.001 |
| 2018** | 1.02 | 1.02-1.02 | <0.001 | 1.01 | 1.01-1.01 | <0.001 |
| 2019** | 1.03 | 1.02-1.03 | <0.001 | 1.01 | 1.01-1.01 | <0.001 |
| 2020** | 1.02 | 1.02-1.02 | <0.001 | 1.02 | 1.01-1.02 | <0.001 |
| 2021** | 1.02 | 1.02-1.03 | <0.001 | 1.01 | 1.01-1.01 | <0.001 |
| **MAP (mmHg)** |  |  |  |  |  |  |
| Overall (2017-2021)* | 1.02 | 1.02-1.02 | <0.001 | 1.01 | 1.01-1.01 | <0.001 |
| 2017** | 1.02 | 1.02-1.02 | <0.001 | 1.01 | 1.01-1.01 | <0.001 |
| 2018** | 1.02 | 1.02-1.02 | <0.001 | 1.01 | 1.01-1.01 | <0.001 |
| 2019** | 1.02 | 1.02-1.03 | <0.001 | 1.01 | 1.01-1.01 | <0.001 |
| 2020** | 1.02 | 1.02-1.02 | <0.001 | 1.01 | 1.01-1.02 | <0.001 |
| 2021** | 1.02 | 1.02-1.03 | <0.001 | 1.01 | 1.01-1.01 | <0.001 |
| **Blood pressure (mmHg)** |  |  |  |  |  |  |
| Overall (2017-2021)* |  |  |  |  |  |  |
| SBP <120 and DBP <80 | Ref. |  |  | Ref. |  |  |
| SBP 120-129 or DBP 80-84 | 1.17 | 1.11-1.22 | <0.001 | 1.09 | 1.05-1.12 | <0.001 |
| SBP 130-139 or DBP 85-89 | 1.35 | 1.30-1.42 | <0.001 | 1.22 | 1.18-1.26 | <0.001 |
| SBP ≥140 or DBP ≥90 | 1.92 | 1.85-2.01 | <0.001 | 1.43 | 1.38-1.48 | <0.001 |
| 2017** |  |  |  |  |  |  |
| SBP <120 and DBP <80 | Ref. |  |  | Ref. |  |  |
| SBP 120-129 or DBP 80-84 | 1.17 | 1.05-1.30 | 0.004 | 1.05 | 0.97-1.14 | 0.197 |
| SBP 130-139 or DBP 85-89 | 1.38 | 1.24-1.53 | <0.001 | 1.15 | 1.06-1.24 | 0.001 |
| SBP ≥140 or DBP ≥90 | 1.88 | 1.70-2.07 | <0.001 | 1.34 | 1.25-1.44 | <0.001 |
| 2018** |  |  |  |  |  |  |
| SBP <120 and DBP <80 | Ref. |  |  | Ref. |  |  |
| SBP 120-129 or DBP 80-84 | 1.15 | 1.04-1.28 | 0.007 | 1.11 | 1.03-1.19 | 0.006 |
| SBP 130-139 or DBP 85-89 | 1.30 | 1.18-1.44 | <0.001 | 1.18 | 1.10-1.27 | <0.001 |
| SBP ≥140 or DBP ≥90 | 1.86 | 1.70-2.03 | <0.001 | 1.36 | 1.27-1.46 | <0.001 |
| 2019** |  |  |  |  |  |  |
| SBP <120 and DBP <80 | Ref. |  |  | Ref. |  |  |
| SBP 120-129 or DBP 80-84 | 1.14 | 1.03-1.26 | 0.011 | 1.01 | 0.94-1.09 | 0.706 |
| SBP 130-139 or DBP 85-89 | 1.35 | 1.23-1.49 | <0.001 | 1.21 | 1.13-1.30 | <0.001 |
| SBP ≥140 or DBP ≥90 | 2.01 | 1.83-2.20 | <0.001 | 1.34 | 1.26-1.44 | <0.001 |
| 2020** |  |  |  |  |  |  |
| SBP <120 and DBP <80 | Ref. |  |  | Ref. |  |  |
| SBP 120-129 or DBP 80-84 | 1.24 | 1.12-1.37 | <0.001 | 1.18 | 1.09-1.28 | <0.001 |
| SBP 130-139 or DBP 85-89 | 1.34 | 1.22-1.48 | <0.001 | 1.34 | 1.23-1.44 | <0.001 |
| SBP ≥140 or DBP ≥90 | 1.91 | 1.74-2.09 | <0.001 | 1.64 | 1.52-1.77 | <0.001 |
| 2021** |  |  |  |  |  |  |
| SBP <120 and DBP <80 | Ref. |  |  | Ref. |  |  |
| SBP 120-129 or DBP 80-84 | 1.13 | 1.01-1.26 | 0.030 | 1.09 | 1.01-1.18 | 0.028 |
| SBP 130-139 or DBP 85-89 | 1.40 | 1.26-1.54 | <0.001 | 1.25 | 1.16-1.34 | <0.001 |
| SBP ≥140 or DBP ≥90 | 1.97 | 1.79-2.17 | <0.001 | 1.50 | 1.40-1.61 | <0.001 |

AOR: adjusted Odds Ratio, *Adjusting for age, regions, body mass index, smoking status, alcohol use, exercise, fasting plasma glucose, total cholesterol, triglyceride and years. **Adjusting for age, regions, body mass index, smoking status, alcohol use, exercise, fasting plasma glucose, total cholesterol, triglyceride.

**Table S2.** Multivariable logistic regression analysis for association between raised blood pressure and elevated aminotransferase in female participants, by year

| **Blood pressure** | **Elevated AST** | | | **Elevated ALT** | | |
| --- | --- | --- | --- | --- | --- | --- |
|  | **AOR** | **95% CI** | ***p*-value** | **AOR** | **95% CI** | ***p*-value** |
| **Female** |  |  |  |  |  |  |
| **SBP (mmHg)** |  |  |  |  |  |  |
| Overall (2017-2021)* | 1.01 | 1.01-1.01 | 0.006 | 1.01 | 1.00-1.01 | <0.001 |
| 2017** | 0.99 | 0.99-101 | 0.471 | 1.01 | 0.99-1.01 | 0.061 |
| 2018** | 1.01 | 1.00-1.02 | 0.023 | 1.01 | 1.00-1.01 | 0.005 |
| 2019** | 1.01 | 1.00-1.02 | 0.006 | 1.01 | 1.00-1.01 | 0.073 |
| 2020** | 1.00 | 1.00-1.01 | 0.421 | 1.00 | 1.00-1.01 | 0.499 |
| 2021** | 1.00 | 1.00-1.01 | 0.302 | 1.00 | 1.00-1.01 | 0.358 |
| **DBP (mmHg)** |  |  |  |  |  |  |
| Overall (2017-2021)* | 1.01 | 1.01-1.01 | 0.001 | 1.01 | 1.01-1.02 | <0.001 |
| 2017** | 1.01 | 0.99-1.02 | 0.281 | 1.01 | 1.00-1.02 | 0.078 |
| 2018** | 1.01 | 1.00-1.03 | 0.014 | 1.01 | 1.00-1.02 | 0.014 |
| 2019** | 1.01 | 1.00-1.02 | 0.028 | 1.01 | 1.00-1.02 | 0.014 |
| 2020** | 1.01 | 1.00-1.01 | 0.276 | 1.01 | 1.01-1.02 | 0.001 |
| 2021** | 1.00 | 0.99-1.01 | 0.745 | 1.02 | 1.01-1.02 | 0.002 |
| **MAP (mmHg)** |  |  |  |  |  |  |
| Overall (2017-2021)* | 1.01 | 1.01-1.01 | 0.001 | 1.01 | 1.01-1.01 | <0.001 |
| 2017** | 1.00 | 0.99-1.01 | 0.740 | 1.01 | 1.00-1.02 | 0.046 |
| 2018** | 1.01 | 1.00-1.03 | <0.001 | 1.01 | 1.01-1.02 | 0.004 |
| 2019** | 1.01 | 1.00-1.02 | 0.008 | 1.01 | 1.00-1.02 | 0.017 |
| 2020** | 1.00 | 1.00-1.01 | 0.282 | 1.01 | 1.00-1.02 | 0.015 |
| 2021** | 1.00 | 0.99-1.02 | 0.483 | 1.01 | 1.00-1.02 | 0.017 |
| **Blood pressure (mmHg)** |  |  |  |  |  |  |
| Overall (2017-2021)* |  |  |  |  |  |  |
| SBP <120 and DBP <80 | Ref. |  |  | Ref. |  |  |
| SBP 120-129 or DBP 80-84 | 1.12 | 0.97-1.30 | 0.135 | 1.22 | 1.09-1.38 | 0.001 |
| SBP 130-139 or DBP 85-89 | 1.34 | 1.16-1.55 | <0.001 | 1.36 | 1.20-1.54 | <0.001 |
| SBP ≥140 or DBP ≥90 | 1.42 | 1.21-1.66 | <0.001 | 1.38 | 1.21-1.57 | <0.001 |
| 2017** |  |  |  |  |  |  |
| SBP <120 and DBP <80 | Ref. |  |  | Ref. |  |  |
| SBP 120-129 or DBP 80-84 | 1.15 | 0.79-1.68 | 0.479 | 1.07 | 0.79-1.45 | 0.655 |
| SBP 130-139 or DBP 85-89 | 1.40 | 0.96-2.02 | 0.078 | 1.67 | 1.25-2.24 | 0.001 |
| SBP ≥140 or DBP ≥90 | 1.13 | 0.76-1.68 | 0.558 | 1.34 | 0.97-1.84 | 0.073 |
| 2018** |  |  |  |  |  |  |
| SBP <120 and DBP <80 | Ref. |  |  | Ref. |  |  |
| SBP 120-129 or DBP 80-84 | 1.36 | 0.96-1.94 | 0.086 | 1.04 | 0.80-1.36 | 0.767 |
| SBP 130-139 or DBP 85-89 | 1.77 | 1.26-2.49 | 0.001 | 1.29 | 0.99-1.67 | 0.060 |
| SBP ≥140 or DBP ≥90 | 1.81 | 1.26-2.60 | 0.001 | 1.45 | 1.10-1.91 | 0.009 |
| 2019** |  |  |  |  |  |  |
| SBP <120 and DBP <80 | Ref. |  |  | Ref. |  |  |
| SBP 120-129 or DBP 80-84 | 1.20 | 0.85-1.68 | 0.302 | 1.14 | 0.89-1.47 | 0.302 |
| SBP 130-139 or DBP 85-89 | 1.48 | 1.06-2.07 | 0.023 | 1.21 | 0.94-1.57 | 0.143 |
| SBP ≥140 or DBP ≥90 | 1.84 | 1.30-2.60 | 0.001 | 1.27 | 0.97-1.67 | 0.302 |
| 2020** |  |  |  |  |  |  |
| SBP <120 and DBP <80 | Ref. |  |  | Ref. |  |  |
| SBP 120-129 or DBP 80-84 | 0.90 | 0.69-1.18 | 0.442 | 1.28 | 0.99-1.64 | 0.055 |
| SBP 130-139 or DBP 85-89 | 1.05 | 0.80-1.38 | 0.706 | 1.22 | 0.93-1.58 | 0.145 |
| SBP ≥140 or DBP ≥90 | 1.16 | 0.87-1.54 | 0.320 | 1.28 | 0.97-1.67 | 0.078 |
| 2021** |  |  |  |  |  |  |
| SBP <120 and DBP <80 | Ref. |  |  | Ref. |  |  |
| SBP 120-129 or DBP 80-84 | 1.23 | 0.86-1.79 | 0.240 | 1.66 | 1.26-2.21 | <0.001 |
| SBP 130-139 or DBP 85-89 | 1.30 | 0.89-1.90 | 0.177 | 1.62 | 1.20-2.20 | 0.002 |
| SBP ≥140 or DBP ≥90 | 1.38 | 0.93-2.03 | 0.110 | 1.62 | 1.19-2.22 | 0.002 |

AOR: adjusted Odds Ratio, *Adjusting for age, regions, body mass index, smoking status, alcohol use, exercise, fasting plasma glucose, total cholesterol, triglyceride and years. **Adjusting for age, regions, body mass index, smoking status, alcohol use, exercise, fasting plasma glucose, total cholesterol, triglyceride.
